# Revision total knee arthroplasty for arthrofibrosis improves range of motion

**DOI:** 10.1007/s00167-023-07353-8

**Published:** 2023-02-21

**Authors:** Zachary A. Rockov, Connor T. Byrne, Kevin T. Rezzadeh, Caleb R. Durst, Andrew I. Spitzer, Guy D. Paiement, Brad L. Penenberg, Sean S. Rajaee

**Affiliations:** Department of Orthopaedic Surgery, Cedars-Sinai Medical Center, 444 South San Vicente Boulevard, Suite 603, Los Angeles, CA 90048 USA

**Keywords:** Arthrofibrosis, Revision total knee arthroplasty, Clinical outcomes, Stiffness, Complications, rTKA, rTKR, ROM, PROMIS

## Abstract

**Purpose:**

Arthrofibrosis after primary total knee arthroplasty (TKA) is a significant contributor to patient dissatisfaction. While treatment algorithms involve early physical therapy and manipulation under anaesthesia (MUA), some patients ultimately require revision TKA. It is unclear whether revision TKA can consistently improve these patient's range of motion (ROM). The purpose of this study was to evaluate ROM when revision TKA was performed for arthrofibrosis.

**Methods:**

A retrospective study of 42 TKA’s diagnosed with arthrofibrosis from 2013 to 2019 at a single institution with a minimum 2-year follow-up was performed. The primary outcome was ROM (flexion, extension, and total arc of motion) before and after revision TKA, and secondary outcomes included patient reported outcomes information system (PROMIS) scores. Categorical data were compared using chi-squared analysis, and paired samples *t* tests were performed to compare ROM at three different times: pre-primary TKA, pre-revision TKA, and post-revision TKA. A multivariable linear regression analysis was performed to assess for effect modification on total ROM.

**Results:**

The patient's pre-revision mean flexion was 85.6 degrees, and mean extension was 10.1 degrees. At the time of the revision, the mean age of the cohort was 64.7 years, the average body mass index (BMI) was 29.8, and 62% were female. At a mean follow-up of 4.5 years, revision TKA significantly improved terminal flexion by 18.4 degrees (*p < *0.001), terminal extension by 6.8 degrees (*p = *0.007), and total arc of motion by 25.2 degrees (*p < *0.001). The final ROM after revision TKA was not significantly different from the patient’s pre-primary TKA ROM (*p = *0.759). PROMIS physical function, depression, and pain interference scores were 39 (SD = 7.72), 49 (SD = 8.39), and 62 (SD = 7.25), respectively.

**Conclusion:**

Revision TKA for arthrofibrosis significantly improved ROM at a mean follow-up of 4.5 years with over 25 degrees of improvement in the total arc of motion, resulting in final ROM similar to pre-primary TKA ROM. PROMIS physical function and pain scores showed moderate dysfunction, while depression scores were within normal limits. While physical therapy and MUA remain the gold standard for the early treatment of stiffness after TKA, revision TKA can improve ROM.

**Level of evidence:**

IV.

## Introduction

Arthrofibrosis after primary total knee arthroplasty (TKA) is a common problem, and prevalence has been reported to be between 1.3 and 5.3% after primary TKA [16, 27]. The percentage of revision arthroplasties done in the United States for arthrofibrosis has been quoted between 4 and 18% [18, 24, 25]. In addition, the burden on the healthcare system is significant, with 27.5% of the 90-day readmissions after TKA being secondary to arthrofibrosis [22]. Stiffness due to arthrofibrosis is not benign and can be debilitating to patients causing pain, fatigue, abnormal gait, difficulty rising from chairs, and difficulty with sexual relations [23]. It has been shown that patients need, on average, about 70 degrees of flexion for normal gait and up to 156 degrees for activities such as kneeling, squatting, and sitting cross-legged [4, 12].

The treatment for arthrofibrosis after TKA ranges from non-operative strategies, including physical therapy and manipulation under anaesthesia (MUA), to more aggressive surgical treatment options such as lysis of adhesions (LOA) or revision arthroplasty [6, 7, 10, 21, 28]. If caught early, preventative measures can be implemented, such as physical therapy, nonsteroidal anti-inflammatory drugs (NSAIDs), passive and active exercises, stretching, and bracing [5]. One of the most common early treatments for arthrofibrosis is performing an MUA. Still, the literature has shown that its utility as a treatment modality is limited when performed more than three months after a TKA [28]. It has been demonstrated that the longer one waits to perform an MUA, the less efficacious the treatment, and the amount of range of motion (ROM) regained is inversely proportional to the length of time between diagnosis and MUA [14]. Other more invasive options include LOA and revision TKA. Arthroscopic LOA can significantly increase ROM with increased functional scores in subsets of patients [6]. However, many patients fail to improve their ROM with an LOA, which leaves revision TKA as a last-stage procedure. There is question in the orthopaedic community as to the ability of revision TKA to improve ROM after TKA when done for stiffness. A recent systematic review has shown that revision arthroplasty for stiffness is efficacious and provides increased ROM and functionality with decreased pain, but this was in a heterogenous patient population with stiffness due to several etiologies [7].

The objective of this study was to evaluate whether revision TKA done specifically for arthrofibrosis can improve ROM at the final follow-up. The hypothesis was that revision TKA for arthrofibrosis would improve ROM compared to the pre-revision TKA ROM.

## Materials and methods

### Study sample

All revision total knee arthroplasties performed for a primary diagnosis of arthrofibrosis or stiffness at a single institution with nine orthopaedic surgeons from 2013 to 2019 were identified retrospectively. Arthrofibrosis was defined as patients having less than 90 degrees of flexion, corresponding to patients with moderate or severe arthrofibrosis, as described by Kalson et al. [15]. Cases revised for more than one cause of revision were excluded to identify patients who underwent a revision for arthrofibrosis specifically. Two arthroplasty-trained orthopaedic surgeons reviewed all knee radiographs to rule out mechanical causes for stiffness. Revisions done for other reasons of stiffness such as infection or history of infection, prior stiffness, patellar maltracking, and component malalignment as described in the international consensus were excluded [15]. Other exclusion criteria included age less than 18 years, periprosthetic fractures, and less than 2 years follow-up.

Forty-two cases of revision TKA for arthrofibrosis were eligible for inclusion in this study, and all data were extracted from the electronic medical record (EMR). The procedure performed and the primary reason for the revision were confirmed by verifying the operative report in the EMR. Patient factors, including sex, age, BMI, and medical comorbidities, were collected. All patients received a weight-based dose of Cefazolin within 1 h of the surgical incision and two post-operative doses every 8 h after surgery. All patients received postoperative deep vein thrombosis prophylaxis based on surgeon preference for 4 weeks. Patients received physical therapy in the hospital postoperatively once a day, focusing on ambulation and knee range of motion, and were discharged to home or rehabilitation based on the physical therapy assessment. There were no deviations in the standard post-operative TKA protocol for any patients.

ROM was collected through the preoperative and postoperative notes based on what was documented in the clinic by the treating physician. ROM was obtained by the treating surgeon based on their visual assessment of flexion and extension and reported as such in the EMR. Patient-reported outcomes measurement information system (PROMIS) scores are collected at every follow-up visit for physical function, depression, and pain interference. PROMIS scores are used at the institution for all patients and are collected at each follow-up visit. The National Institutes of Health designed PROMIS to create a standardized reporting metric to compare patients to the general population with greater precision using *T* scores with a mean and standard deviation of 50 and 10, respectively [3].

The study was conducted in agreement with the ethical standards of the Cedars-Sinai Medical Center institutional research board and with the 1964 Helsinki declaration and its later amendments or comparable ethical standards.

### Data analysis

Descriptive analysis was performed to summarize the data for pre-operative and post-operative range of motion before and after revision TKA. There were three distinct time points where range of motion was assessed in this study: (1) Time point 1 refers to pre-primary TKA (prior to index TKA), (2) time point 2 refers to pre-revision TKA (after index TKA but prior to their revision TKA), (3) time point 3 refers to post-revision TKA (after revision TKA). Mean improvement in range of motion was compared using Student’s *t* tests. Categorical data, including patient demographics, were compared using chi-squared analysis. Paired samples *t* tests were performed to compare ROM at three different times: pre-primary TKA, pre-revision TKA, and post-revision TKA. A multivariable linear regression analysis was performed to assess for effect modification on total ROM by age, gender, laterality, pre-primary ROM, pre-revision ROM, post-revision ROM, and revision type. Outcomes are reported as percentages or as means followed by the standard deviation (SD) in the same units.

The STROBE statement checklist was used in the design of this study and writing of the manuscript [9].

## Results

The mean age of the patients was 64.7 years (SD 7.9), 62% of patients were female, and the mean body mass index (BMI) was 29.8 kg/m^3^ (SD 6.0), Table 1. Procedural laterality was left knee in 38% of cases and right knee in 62.0%. The type of revision performed and the constraint of the revision can be found in Table 2. After revision TKA for arthrofibrosis, there was a significant improvement in range of motion at a mean follow-up of 4.5 (SD:1.7) years relative to pre-revision ROM. On average, extension improved to 3.3 degrees (from 10.1 degrees) (*p = *0.007), flexion improved to 99.2 degrees (from 80.8 degrees) (*p < *0.001), and total arc of motion improved to 95.9 degrees (from 70.7 degrees) (*p < *0.001). Of note, the final range of motion at the final follow-up after revision TKA was not different from the patient’s original pre-operative range of motion prior to their index total knee arthroplasty (*p = *0.759). Table 3 demonstrates the total change in motion between the three-time points (prior to index TKA, prior to revision TKA, and final follow-up after revision TKA). On average, extension was improved by 6.8 degrees after revision, flexion was improved by 18.4 degrees after revision, and arc of motion was improved by 25.2 degrees after revision. PROMIS physical function scores were 39 (SD = 7.72), depression scores were 49 (SD = 8.39), and pain interference scores were 62 (SD = 7.25). These scores represent moderate physical function scores, normal depression levels, and moderate pain interference scores.

**Table 1:** Patient demographics

Sample size	*N*	42
Age (years)	Mean ± SD	64.7 ± 7.9
Sex	Percent female	26 (62.0%)
	Percent male	16 (38.0%)
BMI (kg/m^2^)	Mean ± SD	29.8 ± 6.0
Laterality	Percent left	16 (38.0%)
	Percent right	26 (62.0%)
Follow-up (years)	Mean ± SD	4.5 ± 1.7

*SD* standard deviation; *BMI* body mass index

**Table 2:** Surgical details

Type of revision TKA performed	Number	Percentage of patients (%)
Full revision TKA (all components)	23	54.8
Polyethylene only	12	28.6
Femoral revision	5	11.9
Tibial revision	2	4.8
** Constraint of final revision**		
Hinged prosthesis	1	2.4
Constrained prosthesis	21	50.0
Posterior stabilized prosthesis	16	38.1
Cruciate retaining prosthesis	4	9.5

*TKA* = total knee arthroplasty

**Table 3:** Improvements in range of motion between time points 1 vs 2, 2 vs 3, 1 vs 3

	Extension change	Flexion change	Arc of motion change
Time 1 (pre-primary TKA) to Time 2 (pre-revision)	5.3 (5.4, 10.7) (*p = *0.15)	− 11.2 (96.8, 85.6) (*p < *0.001)	− 16.5 (91.4, 74.9) (*p = *0.015)
Time 2 (pre-revision) to Time 3 (post-revision)	− 6.8 (10.1, 3.3) (*p = *0.007)	18.4 (80.8, 99.2) (*p < *0.001)	25.2 (70.7, 95.9) (*p < *0.001)
Time 1 (pre-primary) to Time 3 (post-revision)	− 1.3 (5.8, 4.5) (*p = *0.583)	0.7 (96.0, 96.7) (*p = *0.915)	2.0 (90.2, 92.2) (*p = *0.759)

TKA = total knee arthroplasty

30.6% of patients in the cohort underwent an MUA procedure prior to the revision, and there were no patients that underwent lysis of adhesions prior to revision. After revision, 21.4% of patients required an MUA, and 2.4% required a lysis of adhesions procedure. 35 of 42 patients did not require further revision (83.3%). Of the seven that did require another revision, 3 had a revision of all components for stiffness, 3 had polyethylene liner exchanges for stiffness, and 1 had an antibiotic spacer placed for a periprosthetic joint infection (PJI). There were 12 polyethylene-only revisions, and of those, nine liners were downsized. All revision arthroplasties had a synovectomy or lysis of adhesions at the time of the revision, regardless of the type of revision.

Subgroup analysis was performed to assess the effects of revision type on improved range of motion, Table 4. Notably, patients that underwent complete revision (femoral and tibial component), representing 54.8% of cases, had significantly improved range of motion in extension, flexion, and total arc of motion. Patients that underwent polyethylene exchange also had a statistically significant improvement in the total arc of motion compared to their pre-revision ROM (*p = *0.012). In a multivariable linear regression model including age, gender, BMI, operative laterality, pre-primary range of motion, and pre-revision range of motion, it showed that a lower pre-revision range of motion was significantly associated with more minor post-revision changes in the total arc of motion (B = -0.648 [95% CI − 0.305 to − 1.035], *p = *0.001).

**Table 4:** Improvement in range of motion based on type of revision total knee arthroplasty from time point 2 (pre-revision) to time point 3 (post-revision)

	Improvement in extension	Improvement in flexion	Improvement in arc of motion
Full revision (both components)	− 8.2 (10.6, 2.4) (*p = *0.011)	19.4 (75.9, 95.3) (*p = *0.001)	27.6 (65.3, 92.9) (*p < *0.001)
Polyethylene revision	− 6.5 (13.4, 6.8) (*p = *0.20)	16.4 (89.6, 105.9) (*p = *0.075)	22.9 (76.2, 99.1) (*p = *0.012)
Femoral revision^a^	− 3.8 (3.8, 0.0) (*p = *0.77)	23.8 (80.0,103.8) (*p = *0.064)	27.5 (76.2, 99.1) (*p = *0.12)
Tibial revision^a^	1.5 (− 1.5, 0.0) (*p = *0.37)	7.5 (87.5, 95.0) (*p = *0.21)	6.0 (89.0, 95.0) (*p = *0.37)

^a^No statistical test completed because only one case in subgroup

## Discussion

The most important finding of the present study is that revision total knee arthroplasty for arthrofibrosis lead to a 25-degree increase in range of motion, equal to the patient's pre-index TKA motion. This is important as post-operative arthrofibrosis continues to be one of the most debilitating complications of total knee arthroplasty and is one of the leading causes of hospital readmission and TKA failure [5]. This problem is estimated to affect 85,000 patients yearly, 25% requiring additional operations to address their deficits in motion [26]. Arthrofibrosis is also one of the leading causes of 90-day hospital readmission and is responsible for up to 18% of revision operations in the immediate postoperative period, thus placing a significant societal and economic burden and having a substantial effect on patient satisfaction [1]. There is a relative paucity of literature describing the specific risk factors of arthrofibrosis; however, factors such as prior knee surgery, smoking, diabetes, and preoperative range of motion are associated with an increased incidence [5]. Although there is no well-defined range of motion criteria to define arthrofibrosis, an arc of motion less than 90 degrees has been shown to lead to significantly lower patient satisfaction [17].

This study demonstrated that revision TKA could significantly improve knee range of motion, limited secondary to post-operative arthrofibrosis. However, patients still have moderate pain interference and physical function PROMIS scores. Prior studies have shown modest improvements in knee range of motion following revision total knee arthroplasty performed to address general stiffness, but PROMIS scores have yet to be reported [7]. Moya-Angeler et al. demonstrated an improvement in patient's flexion contractures from 9.7 to 2.3 degrees, with flexion improving from 81.5 to 94.3 degrees when looking at stiffness after TKA [19]. The only study specific to arthrofibrosis was a recent retrospective review of 46 revision TKAs performed for arthrofibrosis by Rutherford et al., demonstrating that preoperative flexion was increased from 88 degrees to 101, and the mean flexion contracture improved from 11 to 3 degrees [21]. The ROM improvements are similar in this current study, with terminal flexion improving from 80.8 to 99.2 degrees and terminal extension improving from 10.1 to 3.3 degrees. This study additionally showed that revision total knee arthroplasty can return patients to their initial range of motion of pre-index TKA, which has not yet been demonstrated and is important for patient counselling on postoperative expectations.

Interestingly this study found a significant improvement in the total arc of motion when a polyethylene exchange was performed for arthrofibrosis. Notably, all liner exchanges had a lysis of adhesions or synovectomy at the time of surgery, and 75% downsized the polyethylene liner. A few possible explanations for this are that the primary TKA performed may have been tight, leading to difficulty in obtaining ROM in the early postoperative period, which can lead to the formation of arthrofibrosis in addition to slight overstuffing. Another possibility is that when performing the lysis of adhesions, the surgeon could not regain adequate ROM, prompting them to choose to decrease the liner size to gain motion. Other studies have yet to investigate this specifically, and often these patients are excluded from studies looking at revision arthroplasty for the treatment of stiffness [19, 21]. The concept of downsizing the liner, along with a more aggressive lysis of adhesions, allows for more motion but does sacrifice stability. This predicament has led to a recent interest in rotating hinged TKA’s for arthrofibrosis, which is an emerging concept [2, 8, 13, 20].

It should be noted that positive outcomes from undergoing revision arthroplasty should be carefully weighed against the complications associated with the procedure. The reoperation rate associated with revision TKA performed for arthrofibrosis is as high as 49%, although additional literature has demonstrated a wide range of risks [11]. This study showed a 25.6% reoperation rate in the form of MUA (73% of reoperations) and LOA (27% of reoperations). Although this number is high, only 16.7% of patients subsequently required a further revision arthroplasty which is consistent with a recent study looking at revision TKA for arthrofibrosis [21]. Seven patients (16.7%) required revision arthroplasty at final follow-up, including three complete revisions, three polyethylene liner exchanges, and one antibiotic spacer placement. Additionally, numerous studies have shown improvements in range of motion after undergoing manipulations under anaesthesia and arthroscopic lysis of adhesions. Careful consideration should be given to these less invasive procedures before proceeding with revision arthroplasty [10].

This study represents one of the larger cohorts of patients undergoing revision total knee arthroplasty specifically for arthrofibrosis, but it does have limitations. First, the retrospective nature of the review bestows it with inherent design limitations. Second, range of motion measurements were extracted from post-operative clinic visit notes and operative reports. As such, no uniform method was used to measure range of motion as it was based on the surgeon's assessment. In addition, several patients required subsequent MUA, LOA, or another revision to improve motion after their revision TKA, highlighting that revision TKA for arthrofibrosis is not perfect. Lastly, surgical decision-making and performance likely varied amongst the surgeons whose patients were included, and intraoperative assessments of alignment, rotation, and stability were not equal.

## Conclusions

Overall, this study demonstrates that revision TKA improved knee range of motion for patients with postoperative arthrofibrosis. However, patients still have moderate pain interference and physical function PROMIS scores. Choosing to proceed with a revision TKA should be considered an option for patients with arthrofibrosis who have failed conservative measures and less invasive treatment modalities but understand that it does have limitations. In addition, this study found that isolated polyethylene exchange resulted in improvements in ROM approaching that of revision TKA to treat arthrofibrosis.

## Data Availability

Data is not publicly available.
